# Supplementary material for: Sonogenetic control of multiplexed genome regulation and base editing
Source: Nat Commun. 2023 Oct 18;14:6575. doi: 10.1038/s41467-023-42249-8 (PMC10584809; doi:10.1038/s41467-023-42249-8)
Supplement: Supplementary file 1 — Supplementary information [file 41467_2023_42249_MOESM1_ESM.pdf]

Supplemental Information for

Sonogenetic control of multiplexed genome regulation and base editing

Pei Liu[1,2], Josquin Foiret[1], Yinglin Situ[2], Nisi Zhang[1], Aris J K[1,2], Bo Wu[1], Marina N Raie[1], Katherine W Ferrara[1,*], Lei S Qi[2,3,4,*]

Supplementary Figures

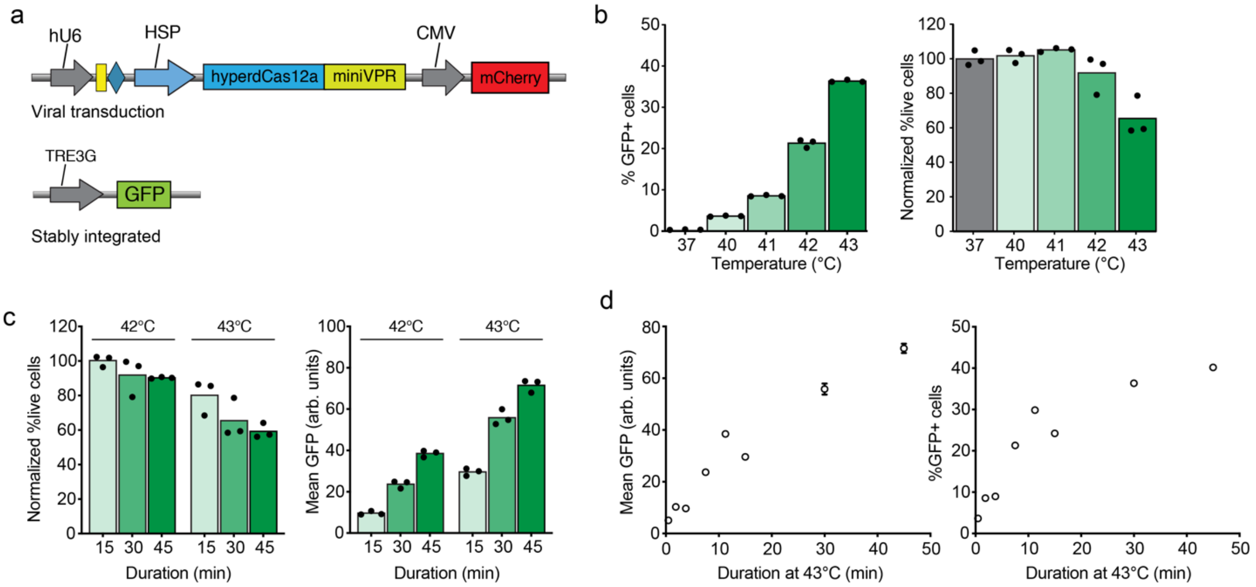

Supplementary Fig. 1: Characterization of how temperature and heat duration affect HSP-hyperdCas12a-mediated gene activation in HEK293T cells.

a, Constructs used for demonstrating GFP reporter activation in HEK cells upon heat treatment. crTet, guide targeting the TRE3G promoter. b, Left: percentage of GFP positive HEK cells expressing hU6-crTet-HSP-hyperdCas12a-miniVPR-CMV-mCherry, measured by flow cytometry 24 hr after cells were treated at various temperatures in a thermal cycler for 30 min. Right: percentage of live cells normalized to the unheated control (i.e., 37˚C). All measured values were normalized to the average percentage of live cells of samples treated at 37°C. c, Left: percentage of live cells normalized to the unheated control (i.e., 37˚C). Right: mean GFP fluorescence intensity measured by flow cytometry when transduced HEK cells were treated at 42˚C or 43˚C for 15, 30, or 45 min. d, Thermal energy input was converted to equivalent minutes at 43˚C according to the Saparato-Dewey equation and the relationship with mean GFP fluorescence intensity (Left) and percentage of GFP positive cells (Right) in HEK cells were plotted. All data are shown for 3 independent replicates. In b, d, %GFP+ cells was calculated as the percentage of GFP-positive cells in the mCherry-positive population (i.e. cells that express HSP-Cas and crTet). Source data in this figure are provided as a Source Data file. Unpaired two-sided t test was used for statistical analysis and p-values are presented in Supplementary Table 3.

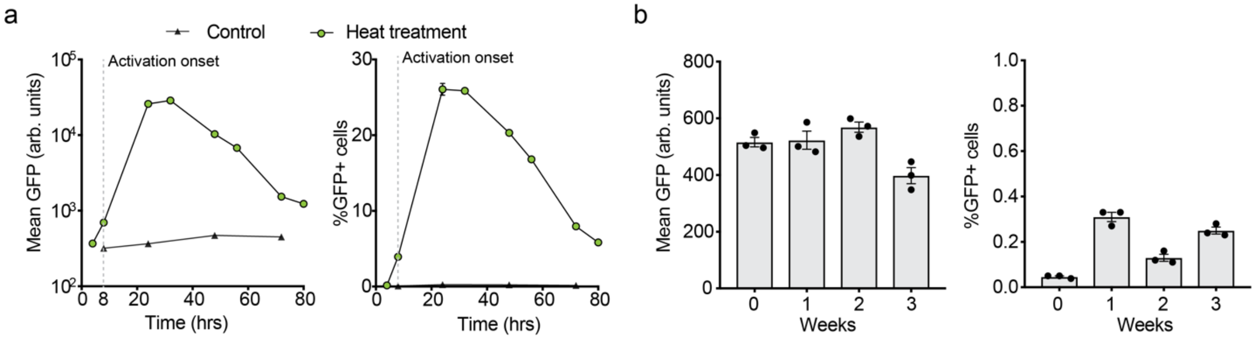

Supplementary Fig. 2: Characterization of time-course heat-induced hyperdCas12a-mediated gene activation in HEK293T cells.

a, Left: mean GFP fluorescence intensity measured by flow cytometry at different time points after cells were treated at 43˚C for 15 min. Right: percentage of GFP positive cells measured at different time points. b, Left: mean GFP fluorescence intensity in HEK cells expressing hU6-crTet-HSP-hyperdCas12a-miniVPR-CMV-mCherry without any thermal treatment, measured over a course of 3 week. Right: percentage of GFP positive cells. %GFP+ cells was calculated as the percentage of GFP-positive cells in the mCherry-positive population (i.e. cells that express HSP-Cas and crTet). All data are shown for 3 independent replicates. Source data in this figure are provided as a Source Data file. Unpaired two-sided t test was used for statistical analysis and p-values are presented in Supplementary Table 3.

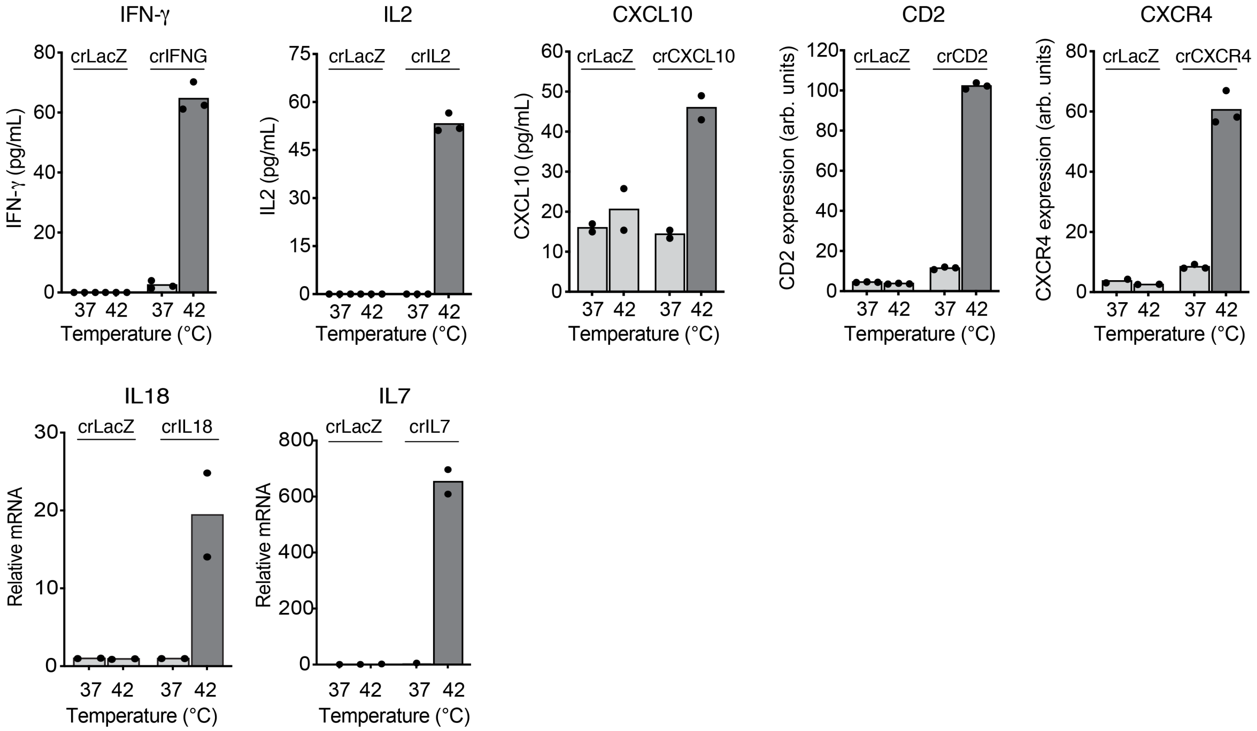

Supplementary Fig. 3: Characterization of heat-induced hyperdCas12a-mediated gene activation for a variety of endogenous genes in HEK293T cells. Endogenous gene activation measured by ELISA (for IFN-γ, IL2, CXCL10) or by immunostaining (for CD2 and CXCR4) or by qPCR (for IL18 and IL7) after cells were transfected with guide RNAs and treated at 42˚C for 30 min. crLacZ: non-targeting guide. All data are shown for 1-3 independent replicates. Source data in this figure are provided as a Source Data file. Unpaired two-sided t test was used for statistical analysis and p-values are presented in Supplementary Table 3.

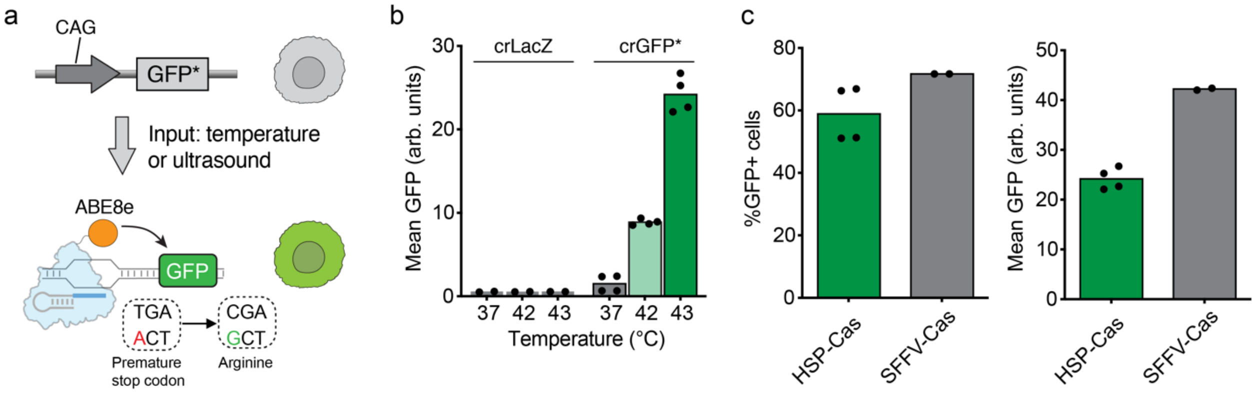

Supplementary Fig. 4: Characterization of heat-induced hyperdCas12a-mediated base editing in HEK293T cells. a, Schematic showing heat-induced base editing using hyperdCas12a. A GFP* reporter containing a premature stop codon (i.e., TGA) was stably integrated in HEK cells. Upon heat or ultrasound treatment, hyperdCas12a fused with adenine base editor is expressed and targets the stop codon with a specific guide RNA to initiate A to G conversion, resulting in full translation and expression of GFP. b, Mean GFP fluorescence intensity measured by flow cytometry when HEK cells were transfected with guide RNAs and treated at 42˚C or 43˚C for 30 min. crLacZ: non-targeting guide; crGFP*: guide targeting the stop codon region in the GFP* reporter. c, Left: percentage of GFP positive cells measured by flow cytometry in heat-treated cells expressing HSP-Cas or cells transfected with a constitutive SFFV-Cas. Both are transfected with guide RNA targeting the stop codon. %GFP+ cells was calculated as the percentage of GFP-positive cells in the mCherry-positive population (i.e. cells that express the guide). Right: mean GFP fluorescence intensity. All data are shown for 2-4 independent replicates. Source data in this figure are provided as a Source Data file. Unpaired two-sided t test was used for statistical analysis and p-values are presented in Supplementary Table 3.

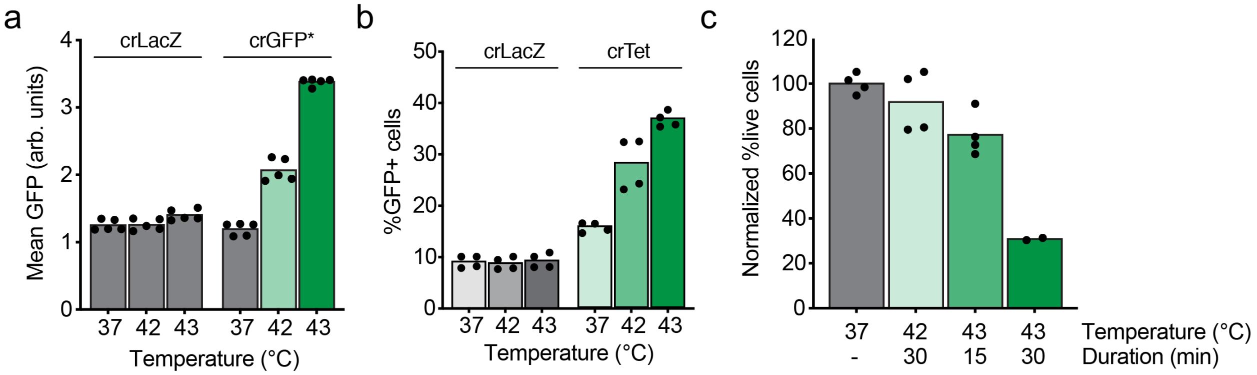

Supplementary Fig. 5: Heat-induced hyperdCas12a for base editing in T cells.

a, Mean GFP fluorescence intensity (i.e., base edited GFP) measured by flow cytometry when transduced Jurkat T cells were treated at 42˚C or 43˚C for 30 min. crLacZ: non-targeting guide; crGFP*: guide targeting the stop codon region in the GFP* reporter. b, Percentage of GFP positive primary T cells after thermal treatment. %GFP+ cells was calculated as the percentage of GFP-positive cells in the mCherry-positive population (i.e. cells that express the guide). c, Percentage of live primary T cells. Primary T cells were heated at 42˚C for 30 min or at 43˚C for 15 or 30 min. All measured values were normalized to the average percentage of live cells of samples treated at 37°C. All data are shown for 2-5 independent replicates. Source data in this figure are provided as a Source Data file. Unpaired two-sided t test was used for statistical analysis and p-values are presented in Supplementary Table 3.

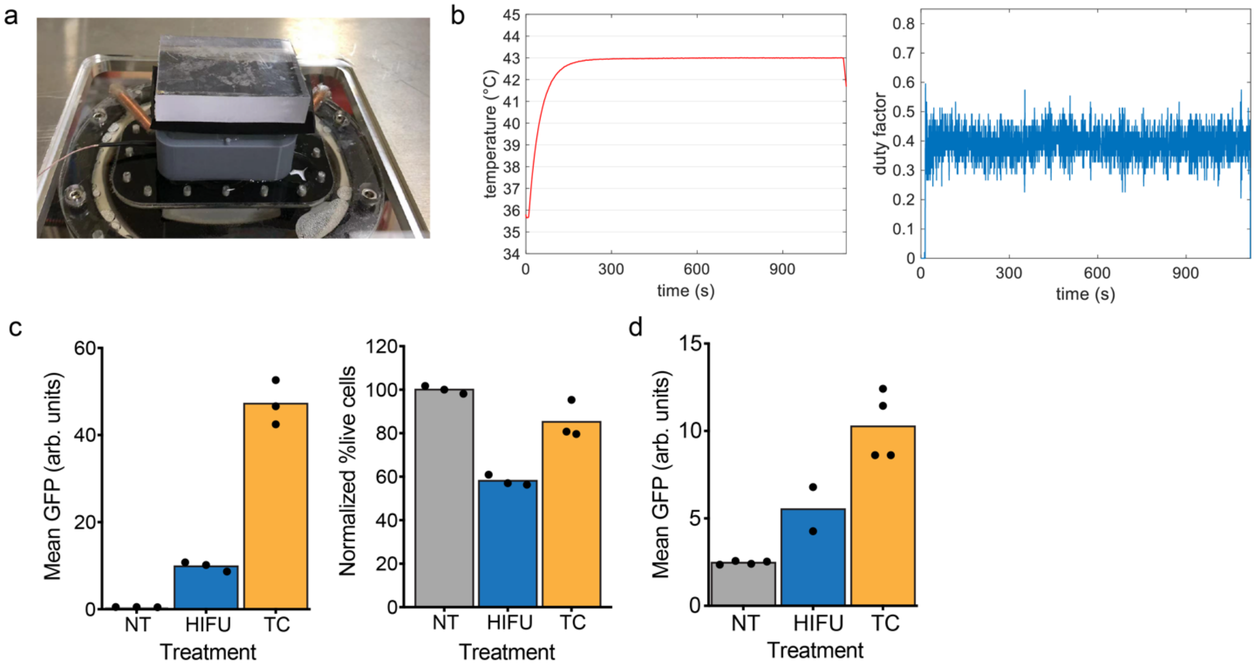

Supplementary Fig. 6. HSP-hyperdCas12a is activated by HIFU *in vitro*.

a, Image of in vitro HIFU setup. Cells in culture media in a 0.6 mL PCR tube were placed in an agar phantom. A needle thermocouple was inserted right next to the bottom of the tube assisted by ultrasound imaging. An ultrasound absorber and weight was placed on top of the PCR tube/phantom. b, Representative trace of temperature (Left) and HIFU output (Right) during 43˚C treatment. c, Left: Mean GFP fluorescence intensity in HEK cells expressing HSP-Cas, crTet and pTRE3G-GFP, measured by flow cytometry 24 hr after cells were treated with HIFU or thermocycler. Heat treatment was done at 43˚C for 15 min. Right: Normalized cell viability after thermal treatment by HIFU or thermocycler at 43°C for 15 min. All measured values were normalized to the average percentage of live cells of samples treated at 37°C. d, Mean GFP fluorescence intensity in HEK cells expressing HSP-Cas, crGFP*, pCAG-GFP*, measured by flow cytometry 24 hr after cells were treated with HIFU. All data are shown for 2-4 independent replicates. Source data in this figure are provided as a Source Data file. Unpaired two-sided t test was used for statistical analysis and p-values are presented in Supplementary Table 3.

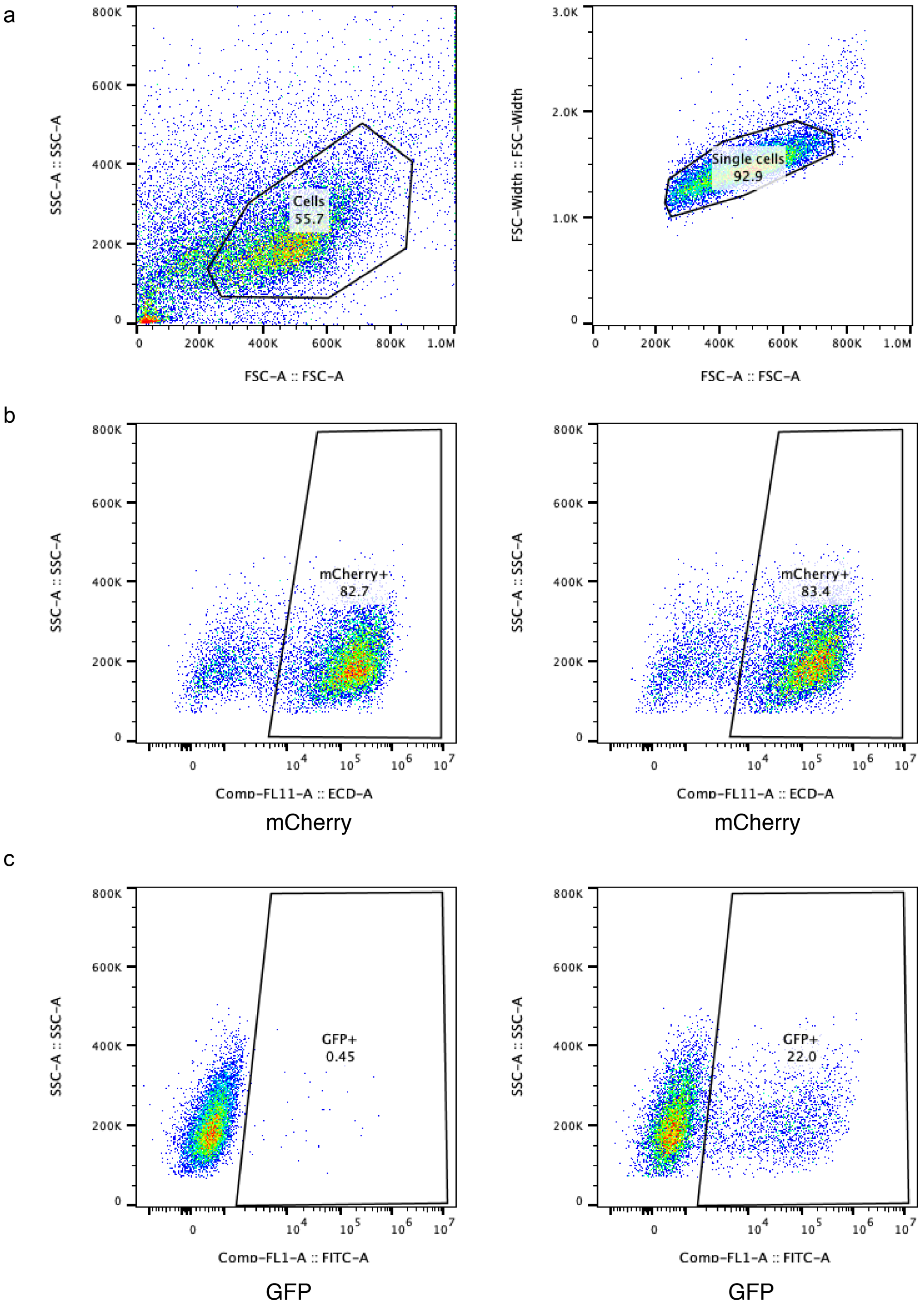

Supplementary Fig. 7. Sample gating strategy for flow cytometry analysis. a, Cells were gated for live cells and then single cells. b, Cells were then gated for expression of fluorescent markers that indicate presence of transfected or transduced constructs. Examples of cells without (left) or with (right) thermal treatment are shown (from Figure 1b and Supplementary Fig. 1a, 37˚C and 42˚C). Both exhibit similar mCherry expression indicating comparable expression of constructs of interest. c, Cells expressing markers were then gated for expression of fluorescent reporter. Examples of cells without (left) or with (right) thermal treatment are shown. Thermal treatment induced strong GFP activation. Specific gating strategy and data analysis for each experiment was stated in figure legends and Methods section.

Supplementary Table 1: Sequences used in this study

Components	Sequence
Truncated HSP promoter	GATCTGAATGGAATGTTCTGGATTGAAGAAAGTGGGAAATGGCCTCAATTCACAAAGTCA<br>CAACCTGATAAAAACCAGTGTGACTTTACTGCCCAGTGAACCCATCTCGTCCTCCAGCCTT<br>TAGGAGGTAGGTTGGACTGGAGCCTGCAGTAGTTTACTCTCCACCTGAGTCCTGGTCTCC<br>AGCTGGGAACCCACTTAGGCCATAGAGAAAAACGCACACTGTGCCTCTCCACCGGGCCTC<br>TGGAGACGAGGCTCCTCGGGGATACAAACAGTGGGGAGAACATGAGGGACATCCCGAC<br>CGTACTCTGCGTCCTCCTTTCCCAGGTGTTGCGTTCTGTCTTGGGCTGAGTGGCGAGGTCT<br>CTCCCGAGTCCCAGGGCCACAGTGCAATGTCACATCTCCTTTGTGGAAAGTGACTGGTAA<br>AGGAGAGAGAACAAAACTGGAGGAACGTAAAGTCTTCAGCCACCTGGTTTAATTTATTCA<br>AGAGTGATTAATCCTAGATGAGAAAAAGAATTGAAATGGATCGGAAAAAAAATGAAAGTGC<br>ATTGGCCGGGAATCGAACCCGGGCCTCCCGCGTGGCAGGCGAGAATTCTACCACTGAACCACCAA<br>TGCTACTGTCAGCTAAAGACCTGCAGTATTGTCTCTTAAAGCTCACTATCTCTGGCCATTCACTAAG<br>GAACCAGGCACCGTCTTAAATCGCGGTTTGGAAAATATTTTGTTCAAGATAAAACTGTTTTAAGAT<br>ATACGTGTATATATCTTATATATCTGTATTCGCATGGTAACATATCTTCGGCCTCCCTGAGCCGCTG<br>GGCTCTCAGCGGCCCTCCAAGGCAGCCCGCAGGCCCCTGTGTGCCTCAGGGATCCGACCTCCCAC<br>AGCCCCGGGGAGACCTTGCCTCTAAAGTTGCTGCTTTTGCAGCCTCTGCCACAACCGCGCGTCCTC<br>AGAGCCAGCCCGGAGGAGCTAGAACCTTCCCCGCATTTCTTTCAGCAGCCTGAGTCAGAGGCGGG<br>CTGGCCTGGCGTAGCCGCCCAGCCTCGCGGCTCATGCCCCGATCTGCCCGAACCTTCTCCCGGGGT<br>CAGCGCCGCGCCGCGCCACCCGGCTGAGTCAGCCCGGGCGGGCGAGAGGCTCTCAACTGGGCGG<br>GAAGGTGCGGGAAGGTGCGGAAAGGTTCGCGAAAGTTCGCGGCGGCGGGGGTCGGGTGAGGC<br>GCAAAAGGATAAAAAGCCCGTGGAAGCGGAGCTGAGCAGATCCGAGCCGGGCTGGCTGCAGAG<br>AAACCGCAGGGAGAGCCTCACTGCTGAGCGCCCCTCGACGGCGGAGCGGCAGCAGCCTCCGTGG<br>CCTCCAGCATCCGACAAGAAGCTTCAGCC
HyperdCas12a<br>-miniVPR<br>hyperdCas12a<br>HA<br>SV40 NLS<br>miniVPR	ATGAGCAAGCTGGAGAAGTTTACAAACTGCTACTCCCTGTCTAAGACCCTGAGGTTCAAGGCCATC<br>CCTGTGGGCAAGACCCAGGAGAACATCGACAATAAGCGGCTGCTGGTGGAGGACGAGAAGAGA<br>GCCGAGGATTATAAGGGCGTGAAGAAGCTGCTGGATCGCTACTATCTGTCTTTTATCAACGACGTG<br>CTGCACAGCATCAAGCTGAAGAATCTGAACAATTACATCAGCCTGTTCCGGAAGAAAACCAGAAC<br>CGAGAAGGAGAATAAGGAGCTGGAGAACCTGGAGATCAATCTGCGGAAGGAGATCGCCAAGGC<br>CTTCAAGGGCAACGAGGGCTACAAGTCCCTGTTTAAGAAGGATATCATCGAGACAATCCTGCCAG<br>AGTTCCTGGACGATAAGGACGAGATCGCCCTGGTGAACAGCTTCAATGGCTTTACCACAGCCTTCA<br>CCGGCTTCTTTCGTAACAGAGAGAATATGTTTTCCGAGGAGGCCAAGAGCACATCCATCGCCTTCA<br>GGTGTATCAACGAGAATCTGACCCGCTACATCTCTAATATGGACATCTTCGAGAAGGTGGACGCCA<br>TCTTTGATAAGCACGAGGTGCAGGAGATCAAGGAGAAGATCCTGAACAGCGACTATGATGTGGA<br>GGATTTCTTTGAGGGCGAGTTCTTTAACTTTGTGCTGACACAGGAGGGCATCCGCGTGTATAACGC<br>CATCATCGGCGGCTTCGTGACCGAGAGCGGCGAGAAGATCAAGGGCCTGAACGAGTACATCAAC<br>CTGTATAATCAGAAAACCAAGCAGAAGCTGCCTAAGTTTAAGCCACTGTATAAGCAGGTGCTGAG<br>CGATCGGGAGTCTCTGAGCTTCTACGGCAGGGGCTATACATCCGATGAGGAGGTGCTGGAGGTG<br>TTTAGAAACACCCTGAACAAGAACAGCGAGATCTTCAGCTCCATCAAGAAGCTGGAGAAGCTGTT<br>CAAGAATTTTGACGAGTACTCTAGCGCCGGCATCTTTGTGAAGAACGGCCCCGCCATCAGCACAAT<br>CTCCAAGCGTATCTTCGGCGAGTGGAACGTGATCCGGGACAAGTGGAATGCCGAGTATGACGATA<br>TCCACCTGAAGAAGAAGGCCGTGGTGACCGAGAAGTACGAGGACGATCGGAGAAAGTCCTTCAA<br>GAAGATCGGCTCCTTTTCTCTGGAGCAGCTGCAGGAGTACGCCGACGCCGATCTGTCTGTGGTGG

	AGAAGCTGAAGGAGATCATCATCCAGAAGGTGGATGAGATCTACAAGGTGTATGGCTCCTCTGAG<br>AAGCTGTTCGACGCCGATTTTGTGCTGGAGAAGAGCCTGAAGAAGAACGACGCCGTGGTGGCCAT<br>CATGAAGGACCTGCTGGATTCTGTGAAGAGCTTCGAGAATTACATCAAGGCCTTCTTTGGCGAGG<br>GCAAGGAGACAAACAGGGACGAGTCCTTCTATGGCGATTTTGTGCTGGCCTACGACATCCTGCTG<br>AAGGTGGACCACATCTACGATGCCATCCGCAATTATGTGACCCAGAAGCCCTACTCTAAGGATAAG<br>TTCAAGCTGTATTTTCAGAACCCTCAGTTCATGGGCGGCTGGGACAAGGATAAGGAGACAGACTA<br>TCGGGCCACCATCCTGAGATACGGCTCCAAGTACTATCTGGCCATCATGGATAAGAAGTACGCCAA<br>GTGCCTGCAGAAGATCGACAAGGACGATGTGAACGGCAATTACGAGAAGATCAACTATAAGCTG<br>CTGCCCGGCCCTAATAAGATGCTGCCAAAGGTGTTCTTTTCTAAGAAGTGGATGGCCTACTATAAC<br>CCCAGCGAGGACATCCAGAAGATCTACAAGAATGGCACATTCAAGAAGGGCGATATGTTTAACCT<br>GAATGACTGTCACAAGCTGATCGACTTCTTTAAGGATAGCATCTCCCGGTATCCAAAGTGGTCCAA<br>TGCCTACGATTTCAACTTTTCTGAGACAGAGAAGTATAAGGACATCGCCGGCTTTTACAGAGAGGT<br>GGAGGAGCAGGGCTATAAGGTGAGCTTCGAGTCTGCCAGCAAGAAGGAGGTGGATAAGCTGGT<br>GGAGGAGGGCAAGCTGTATATGTTCCAGATCTATAACAAGGACTTTTCCGATAAGTCTCACGGCA<br>CACCCAATCTGCACACCATGTACTTCAAGCTGCTGTTTGACGAGAACAATCACGGACAGATCAGGC<br>TGAGCGGAGGAGCAGAGCTGTTCATGAGGCGCGCCTCCCTGAAGAAGGAGGAGCTGGTGGTGCA<br>CCCAGCCAACTCCCCTATCGCCAACAAGAATCCAGATAATCCCAAGAAAACCACAACCCTGTCCTA<br>CGACGTGTATAAGGATAAGAGGTTTTCTGAGGACCAGTACGAGCTGCACATCCCAATCGCCATCA<br>ATAAGTGCCCCAAGAACATCTTCAAGATCAATACAGAGGTGCGCGTGCTGCTGAAGCACGACGAT<br>AACCCCTATGTGATCGGCATCGCCAGGGGCGAGCGCAATCTGCTGTATATCGTGGTGGTGGACGG<br>CAAGGGCAACATCGTGGAGCAGTATTCCCTGAACGAGATCATCAACAACTTCAACGGCATCAGGA<br>TCAAGACAGATTACCACTCTCTGCTGGACAAGAAGGAGAAGGAGAGGTTCGAGGCCCGCCAGAA<br>CTGGACCTCCATCGAGAATATCAAGGAGCTGAAGGCCGGCTATATCTCTCAGGTGGTGCACAAGA<br>TCTGCGAGCTGGTGGAGAAGTACGATGCCGTGATCGCCCTGGAGGACCTGAACTCTGGCTTTAAG<br>AATAGCCGCGTGAAGGTGGAGAAGCAGGTGTATCAGAAGTTCGAGAAGATGCTGATCGATAAGC<br>TGAACTACATGGTGGACAAGAAGTCTAATCCTTGTGCAACAGGCGGCGCCCTGAAGGGCTATCAG<br>ATCACCAATAAGTTCGAGAGCTTTAAGTCCATGTCTACCCAGAACGGCTTCATCTTTTACATCCCTG<br>CCTGGCTGACATCCAAGATCGATCCATCTACCGGCTTTGTGAACCTGCTGAAAACCAAGTATACCA<br>GCATCGCCGATTCCAAGAAGTTCATCAGCTCCTTTGACAGGATCATGTACGTGCCCGAGGAGGATC<br>TGTTCGAGTTTGCCCTGGACTATAAGAACTTCTCTCGCACAGACGCCGATTACATCAAGAAGTGGA<br>AGCTGTACTCCTACGGCAACCGGATCAGAATCTTCCGGAATCCTAAGAAGAACAACGTGTTCGACT<br>GGGAGGAGGTGTGCCTGACCAGCGCCTATAAGGAGCTGTTCAACAAGTACGGCATCAATTATCAG<br>CAGGGCGATATCAGAGCCCTGCTGTGCGAGCAGTCCGACAAGGCCTTCTACTCTAGCTTTATGGCC<br>CTGATGAGCCTGATGCTGCAGATGCGGAACAGCATCACAGGCCGCACCGACGTGGATTTTCTGAT<br>CAGCCCTGTGAAGAACTCCGACGGCATCTTCTACGATAGCCGGAACTATGAGGCCCAGGAGAATG<br>CCATCCTGCCAAAGAACGCCGACGCCAATGGCGCCTATAACATCGCCAGAAAGGTGCTGTGGGCC<br>ATCGGCCAGTTCAAGAAGGCCGAGGACGAGAAGCTGGATAAGGTGAAGATCGCCATCTCTAACA<br>AGGAGTGGCTGGAGTACGCCCAGACCAGCGTGAAGCACGGATCCTATCCCTATGACGTGCCCGAT<br>TATGCCAGCCTGGGCAGCGGCTCCCCCAAGAAAAAACGCAAGGTGGAAGATCCTAAGAAAAAGC<br>GGAAAGTGGACGGCATTGGTAGTGGGAGCAACGGCAGCAGCCTCGATGCTTTAGACGATTTTGA<br>CTTAGATATGCTTGGTTCAGACGCGTTAGACGACTTCGACCTAGACATGTTAGGCTCAGATGCATT<br>GGACGACTTCGATTTAGATATGTTGGGCTCCGATGCCCTAGATGACTTTGATCTAGATATGCTAGG<br>TAGTCCCAAAAAGAAGAGGAAAGTGGGATCCGTCCTTCCGCAAGCGCCTGCCCCAGCGCCTGCAC<br>CGGCAATGGTGTCCGCCCTCGCACAGGCCCCTGCGCCCGTCCCCGTGCTCGCGCCTGGACCGCCCC<br>AGGCGGTCGCTCCACCGGCTCCGAAGCCGACGCAGGCCGGAGAGGGAACACTCTCCGAAGCACT<br>TCTTCAACTCCAGTTTGATGACGAGGATCTTGGAGCACTCCTTGGAAACTCGACAGACCCTGCGGT<br>GTTTACCGACCTCGCGTCAGTAGATAACTCCGAATTTCAGCAGCTTTTGAACCAGGGTATCCCGGT<br>CGCGCCACATACAACGGAGCCCATGTTGATGGAATACCCCGAAGCAATCACGAGACTTGTGACGG

	GAGCGCAGCGGCCTCCCGATCCCGCACCCGCACCTTTGGGGGCACCTGGCCTCCCTAACGGACTTTTGAGCGGCGACGAGGATTTCTCCTCCATCGCCGATATGGATTTCTCAGCCTTGCTGTCAGGCTCTGGCAGCGGCAGCGACCTTTCCCATCCGCCCCCAAGGGGCCATCTGGATGAGCTGACAACCACACTTGAGTCCATGACCGAGGATCTGAACCTGGACTCACCCCTGACCCCGGAATTGAACGAGATTCTGGATACCTTCCTGAACGACGAGTGCCTCTTGCATGCCATGCATATCAGCACAGGACTGTCCATCTTCGACACATCTCTGTTT
ABE8e-hyperdCas12a<br>NLS<br>ABE8e<br>hyperdCas12a	ATGAAACGGACAGCCGACGGAAGCGAGTTCGAGTCACCAAAGAAGAAGCGGAAAGTCTCTGAGGTGGAGTTTTCCCACGAGTACTGGATGAGACATGCCCTGACCCTGGCCAAGAGGGCACGGGATGAGAGGGAGGTGCCTGTGGGAGCCGTGCTGGTGCTGAACAATAGAGTGATCGGCGAGGGCTGGAACAGAGCCATCGGCCTGCACGACCCAACAGCCCATGCCGAAATTATGGCCCTGAGACAGGGCGGCCTGGTCATGCAGAACTACAGACTGATTGACGCCACCCTGTACGTGACATTCGAGCCTTGCGTGATGTGCGCCGGCGCCATGATCCACTCTAGGATCGGCCGCGTGGTGTTTGGCGTGAGGAACTCAAAAAGAGGCGCCGCAGGCTCCCTGATGAACGTGCTGAACTACCCCGGCATGAATCACCGCGTCGAAATTACCGAGGGAATCCTGGCAGATGAATGTGCCGCCCTGCTGTGCGATTTCTATCGGATGCCTAGACAGGTGTTCAATGCTCAGAAGAAGGCCCAGAGCTCCATCAACTCTGGAGGATCTAGCGGAGGATCCTCTGGCAGCGAGACACCAGGAACAAGCGAGTCAGCAACACCAGAGAGCAGTGGCGGCAGCAGCGGCGGCAGCAGCAAGCTGGAGAAGTTTACAAACTGCTACTCCCTGTCTAAGACCCTGAGGTTCAAGGCCATCCCTGTGGGCAAGACCCAGGAGAACATCGACAATAAGCGGCTGCTGGTGGAGGACGAGAAGAGAGCCGAGGATTATAAGGGCGTGAAGAAGCTGCTGGATCGCTACTATCTGTCTTTTATCAACGACGTGCTGCACAGCATCAAGCTGAAGAATCTGAACAATTACATCAGCCTGTTCCGGAAGAAAACCAGAACCGAGAAGGAGAATAAGGAGCTGGAGAACCTGGAGATCAATCTGCGGAAGGAGATCGCCAAGGCCTTCAAGGGCAACGAGGGCTACAAGTCCCTGTTTAAGAAGGATATCATCGAGACAATCCTGCCAGAGTTCCTGGACGATAAGGACGAGATCGCCCTGGTGAACAGCTTCAATGGCTTTACCACAGCCTTCACCGGCTTCTTTCGGAACAGAGAGAATATGTTTTCCGAGGAGGCCAAGAGCACATCCATCGCCTTCAGGTGTATCAACGAGAATCTGACCCGCTACATCTCTAATATGGACATCTTCGAGAAGGTGGACGCCATCTTTGATAAGCACGAGGTGCAGGAGATCAAGGAGAAGATCCTGAACAGCGACTATGATGTGGAGGATTTCTTTGAGGGCGAGTTCTTTAACTTTGTGCTGACACAGGAGGGCATCCGCGTGTATAACGCCATCATCGGCGGCTTCGTGACCGAGAGCGGCGAGAAGATCAAGGGCCTGAACGAGTACATCAACCTGTATAATCAGAAAACCAAGCAGAAGCTGCCTAAGTTTAAGCCACTGTATAAGCAGGTGCTGAGCGATCGGGAGTCTCTGAGCTTCTACGGCCGGGGCTATACATCCGATGAGGAGGTGCTGGAGGTGTTTAGAAACACCCTGAACAAGAACAGCGAGATCTTCAGCTCCATCAAGAAGCTGGAGAAGCTGTTCAAGAATTTTGACGAGTACTCTAGCGCCGGCATCTTTGTGAAGAACGGCCCCGCCATCAGCACAATCTCCAAGCGTATCTTCGGCGAGTGGAACGTGATCCGGGACAAGTGGAATGCCGAGTATGACGATATCCACCTGAAGAAGAAGGCCGTGGTGACCGAGAAGTACGAGGACGATCGGAGAAAGTCCTTCAAGAAGATCGGCTCCTTTTCTCTGGAGCAGCTGCAGGAGTACGCCGACGCCGATCTGTCTGTGGTGGAGAAGCTGAAGGAGATCATCATCCAGAAGGTGGATGAGATCTACAAGGTGTATGGCTCCTCTGAGAAGCTGTTCGACGCCGATTTTGTGCTGGAGAAGAGCCTGAAGAAGAACGACGCCGTGGTGGCCATCATGAAGGACCTGCTGGATTCTGTGAAGAGCTTCGAGAATTACATCAAGGCCTTCTTTGGCGAGGGCAAGGAGACAAACAGGGACGAGTCCTTCTATGGCGATTTTGTGCTGGCCTACGACATCCTGCTGAAGGTGGACCACATCTACGATGCCATCCGCAATTATGTGACCCAGAAGCCCTACTCTAAGGATAAGTTCAAGCTGTATTTTCAGAACCCTCAGTTCATGGGCGGCTGGGACAAGGATAAGGAGACAGACTATCGGGCCACCATCCTGAGATACGGCTCCAAGTACTATCTGGCCATCATGGATAAGAAGTACGCCAAGTGCCTGCAGAAGATCGACAAGGACGATGTGAACGGCAATTACGAGAAGATCAACTATAAGCTGCTGCCCGGCCCTAATAAGATGCTGCCAAAGGTGTTCTTTTCTAAGAAGTGGATGGCCTACTATAACCCCAGCGAGGACATCCAGAAGATCTACAAGAATGGCACATTCAAGAAGGGCGATATGTTTAACCTGAATGACTGTCACAAGCTGATCGACTTCTTTAAGGATAGCATCTCCCGGTATCCAAAGTGGTCCAATGCCTACGATTTCAACTTTTCTGAGACAGAGAAGTATAAGGACATCGCCGGCTTTTACAGAGAGGTGGAGGAGCAGGGCTATAAGGTGAGCTTCGAGTCTGCCAGCAAGAAGGAGGTGGATAAGCT

	GGTGGAGGAGGGCAAGCTGTATATGTTCCAGATCTATAACAAGGACTTTTCCGATAAGTCTCACG<br>GCACACCCAATCTGCACACCATGTACTTCAAGCTGCTGTTTGACGAGAACAATCACGGACAGATCA<br>GGCTGAGCGGAGGAGCAGAGCTGTTCATGAGGCGCGCCTCCCTGAAGAAGGAGGAGCTGGTGG<br>TGCACCCAGCCAACTCCCCTATCGCCAACAAGAATCCAGATAATCCCAAGAAAACCACAACCCTGT<br>CCTACGACGTGTATAAGGATAAGAGGTTTTCTGAGGACCAGTACGAGCTGCACATCCCAATCGCC<br>ATCAATAAGTGCCCCAAGAACATCTTCAAGATCAATACAGAGGTGCGCGTGCTGCTGAAGCACGA<br>CGATAACCCCTATGTGATCGGCATCGCCAGGGGCGAGCGCAATCTGCTGTATATCGTGGTGGTGG<br>ACGGCAAGGGCAACATCGTGGAGCAGTATTCCCTGAACGAGATCATCAACAACTTCAACGGCATC<br>AGGATCAAGACAGATTACCACTCTCTGCTGGACAAGAAGGAGAAGGAGAGGTTCGAGGCCCGCC<br>AGAACTGGACCTCCATCGAGAATATCAAGGAGCTGAAGGCCGGCTATATCTCTCAGGTGGTGCAC<br>AAGATCTGCGAGCTGGTGGAGAAGTACGATGCCGTGATCGCCCTGGAGGACCTGAACTCTGGCTT<br>TAAGAATAGCCGCGTGAAGGTGGAGAAGCAGGTGTATCAGAAGTTCGAGAAGATGCTGATCGAT<br>AAGCTGAACTACATGGTGGACAAGAAGTCTAATCCTTGTGCAACAGGCGGCGCCCTGAAGGGCTA<br>TCAGATCACCAATAAGTTCGAGAGCTTTAAGTCCATGTCTACCCAGAACGGCTTCATCTTTTACATC<br>CCTGCCTGGCTGACATCCAAGATCGATCCATCTACCGGCTTTGTGAACCTGCTGAAAACCAAGTAT<br>ACCAGCATCGCCGATTCCAAGAAGTTCATCAGCTCCTTTGACAGGATCATGTACGTGCCCGAGGAG<br>GATCTGTTCGAGTTTGCCCTGGACTATAAGAACTTCTCTCGCACAGACGCCGATTACATCAAGAAG<br>TGGAAGCTGTACTCCTACGGCAACCGGATCAGAATCTTCCGGAATCCTAAGAAGAACAACGTGTTC<br>GACTGGGAGGAGGTGTGCCTGACCAGCGCCTATAAGGAGCTGTTCAACAAGTACGGCATCAATTA<br>TCAGCAGGGCGATATCAGAGCCCTGCTGTGCGAGCAGTCCGACAAGGCCTTCTACTCTAGCTTTAT<br>GGCCCTGATGAGCCTGATGCTGCAGATGCGGAACAGCATCACAGGCCGCACCGACGTGGATTTTC<br>TGATCAGCCCTGTGAAGAACTCCGACGGCATCTTCTACGATAGCCGGAACTATGAGGCCCAGGAG<br>AATGCCATCCTGCCAAAGAACGCCGACGCCAATGGCGCCTATAACATCGCCAGAAAGGTGCTGTG<br>GGCCATCGGCCAGTTCAAGAAGGCCGAGGACGAGAAGCTGGATAAGGTGAAGATCGCCATCTCT<br>AACAAGGAGTGGCTGGAGTACGCCCAGACCAGCGTGAAGCACAAAAGGCCGGCGGCCACGAAA<br>AAGGCCGGCCAGGCAAAAAAGAAAAAG
GFP*<br>premature stop	ATGAGCAAAGGAGAAGAACTTTTCACTGGAGTTGTCCCAATTCTTGTTGAATTAGATGGT<br>GATGTTAATGGGCACAAATTTTCTGTCCGTGGAGAGGGTGAAGGTGATGCTACAAACGG<br>AAAACTCACCCTTAAATTTATTTGCACTACTGGAAAACTACCTGTTCCGTGGCCAACACTT<br>GTCACTACTCTGACCTATGGTGTTCAATGCTTTTCCCGTTATCCGGATCACATGAAACGGC<br>ATGACTTTTTCAAGAGTGCCATGCCCGAAGGTTATGTACAGGAACGCACTATATCTTTCAAAGA<br>TGACGGGACCTACAAGACGCGTGCTGAAGTCAAGTTTGAAGGTGATACCCTTGTTAACTGAATCG<br>AGTTAAAGGGTATTGATTTTAAAGAAGATGGAAACATTCTTGGACACAAACTCGAGTACAACTTTA<br>ACTCACACAATGTATACATCACGGCAGACAAACAAAAGAATGGAATCAAAGCTAACTTCAAAATTC<br>GCCACAACGTTGAAGATGGTTCCGTTCAACTAGCAGACCATTATCAACAAAATACTCCAATTGGCG<br>ATGGCCCTGTCCTTTTACCAGACAACCATTACCTGTCGACACAATCTGTCCTTTCGAAAGATCCCAA<br>CGAAAAGCGTGACCACATGGTCCTTCTTGAGTTTGTAACTGCTGCTGGGATTACACATGGCATGGA<br>TGAGCTCTACAAA
TRE3G promoter<br>TetO repeat	TTACTCCCTATCAGTGATAGAGAACGTATGAAGAGTTTACTCCCTATCAGTGATAGAGAACGTATG<br>CAGACTTTACTCCCTATCAGTGATAGAGAACGTATAAGGAGTTTACTCCCTATCAGTGATAGAGAA<br>CGTATGACCAGTTTACTCCCTATCAGTGATAGAGAACGTATCTACAGTTTACTCCCTATCAGTGATA<br>GAGAACGTATATCCAGTTTACTCCCTATCAGTGATAGAGAACGTATGTCGAGGTAGGCGTGTACG<br>GTGGGCGCCTATAAAAGCAGAGCTCGTTTAGTGAACCGTCAGATCGCCTGGAGCAATTCCACAAC<br>ACTTTTGTCTTATACTT

Supplementary Table 2: crRNA used in this study

crRNA	Spacer sequence
crTet	ctccctatcagtgatagagaacg
crIFNg	agatgagatggtgacagataggc
crIL2	atacagaaggcgttaattgcatg<br>ttacattagcccacacttaggtg
crCXCR4	gcaaggatggacgcgccacagag<br>cgggtggtcggtagtgagtccgg
crCD2	tgttactgtaaaagatgtaaaga
crCXCL10	ctaagtcaactgtaatgccctt<br>cctccctaattctgattggata
crFOXQ1	actactacagaagcacatacgc<br>ccgccgcgctgcagtgtggcct
crIL18	tgctgaagtgtgaccaggaagtc
crIL7	aaagacgacttggcatcgtccac
crLacZ	cgaatacgcccacgcgatggg
crGFP*	actcgattcagttaacaagggta

Supplementary Table 3: Statistical analysis

Figure	Condition 1	Condition 2	p-value
Fig. 1b	37°C	40°C	0.0001
	37°C	41°C	<0.0001
	37°C	42°C	<0.0001
	37°C	43°C	<0.0001
Fig. 1c (IFN-γ)	37°C	42°C	<0.0001
Fig. 1c (CD2)	37°C	42°C	<0.0001
Fig. 2a (IFN-γ)	- (heat)	+ (heat)	<0.0001
Fig. 2a (IL2)	- (heat)	+ (heat)	<0.0001
Fig. 2b (IL18)	- (heat)	+ (heat)	<0.0001
Fig. 2b (IL7)	- (heat)	+ (heat)	<0.0001
Fig. 2b (IFNG)	- (heat)	+ (heat)	<0.0001
Fig. 2b (IL2)	- (heat)	+ (heat)	<0.0001
Fig. 2c (crGFP*)	37°C	42°C	<0.0001
Fig. 2c (crGFP*)	37°C	43°C	<0.0001
Fig. 3a (IFN-γ)	37°C	42°C	0.0011
Fig. 3a (IL2)	37°C	42°C	0.0008
Fig. 3b (crGFP*)	37°C	42°C	<0.0001
Fig. 3b (crGFP*)	37°C	43°C	<0.0001
Fig. 3c (crTet)	37°C	42°C	0.003
	37°C	43°C	<0.0001
Fig. 3d (crFOXQ1)	37°C	43°C	0.0057
Fig. 4b	NT	HIFU	<0.0001
	NT	TC	<0.0001
Fig. 4c (IL18)	NT	HIFU	0.0151
Fig. 4c (IL7)	NT	HIFU	0.0235
Fig. 4c (IFNG)	NT	HIFU	0.0199
Fig. 4c (IL2)	NT	HIFU	0.0003
Fig. 4d	NT	HIFU	0.0096
	NT	TC	<0.0001
Fig. 5d	NT	HIFU	0.0005
Supplementary Fig. 1b (% GFP+ cells)	37°C	40°C	<0.0001
	37°C	41°C	<0.0001
	37°C	42°C	<0.0001
	37°C	43°C	<0.0001
Supplementary Fig. 1b (% live cells)	37°C	40°C	0.6227
	37°C	41°C	0.1167
	37°C	42°C	0.305
	37°C	43°C	0.0081
Supplementary Fig. 1c	37°C	42°C 15min	0.9433

(% live cells)	37°C	42°C 30min	0.305
	37°C	42°C 45min	0.0186
	37°C	43°C 15min	0.0361
	37°C	43°C 30min	0.0081
	37°C	43°C 45min	0.0003
Supplementary Fig. 1c (mean GFP)	37°C	42°C 15min	<0.0001
	37°C	42°C 30min	<0.0001
	37°C	42°C 45min	<0.0001
	37°C	43°C 15min	<0.0001
	37°C	43°C 30min	<0.0001
	37°C	43°C 45min	<0.0001
Supplementary Fig. 3 (crIFNG)	37°C	42°C	<0.0001
Supplementary Fig. 3 (crIL2)	37°C	42°C	<0.0001
Supplementary Fig. 3 (crCXCL10)	37°C	42°C	0.0099
Supplementary Fig. 3 (crCD2)	37°C	42°C	<0.0001
Supplementary Fig. 3 (crCXCR4)	37°C	42°C	<0.0001
Supplementary Fig. 3 (crIL18)	37°C	42°C	<0.0001
Supplementary Fig. 3 (crIL7)	37°C	42°C	<0.0001
Supplementary Fig. 4b (crGFP*)	37°C	42°C	<0.0001
Supplementary Fig. 4b (crGFP*)	37°C	43°C	<0.0001
Supplementary Fig. 5a (crGFP*)	37°C	42°C	<0.0001
Supplementary Fig. 5a (crGFP*)	37°C	43°C	<0.0001
Supplementary Fig. 5b (crTet)	37°C	42°C	0.003
Supplementary Fig. 5b (crTet)	37°C	43°C	<0.0001
Supplementary Fig. 6c (mean GFP)	NT	HIFU	<0.0001
Supplementary Fig. 6c (mean GFP)	NT	TC	<0.0001
Supplementary Fig. 6d	NT	HIFU	0.0169

Supplementary Fig. 6d	NT	TC	0.0002
