# Supplementary material for: Sonogenetic control of multiplexed genome regulation and base editing
Source: Nat Commun. 2023 Oct 18;14:6575. doi: 10.1038/s41467-023-42249-8 (PMC10584809; doi:10.1038/s41467-023-42249-8)
Supplement: Supplementary file 2 — Reporting Summary [file 41467_2023_42249_MOESM2_ESM.pdf]

nature portfolio

Corresponding author(s): Lei S Qi, Katherine W Ferrara

Last updated by author(s): Sep 26, 2023

Reporting Summary

Nature Portfolio wishes to improve the reproducibility of the work that we publish. This form provides structure for consistency and transparency in reporting. For further information on Nature Portfolio policies, see our Editorial Policies and the Editorial Policy Checklist.

Statistics

For all statistical analyses, confirm that the following items are present in the figure legend, table legend, main text, or Methods section.

n/a	Confirmed	
☐	☒	The exact sample size (*n*) for each experimental group/condition, given as a discrete number and unit of measurement
☐	☒	A statement on whether measurements were taken from distinct samples or whether the same sample was measured repeatedly
☐	☒	The statistical test(s) used AND whether they are one- or two-sided<br>*Only common tests should be described solely by name; describe more complex techniques in the Methods section.*
☒	☐	A description of all covariates tested
☒	☐	A description of any assumptions or corrections, such as tests of normality and adjustment for multiple comparisons
☐	☒	A full description of the statistical parameters including central tendency (e.g. means) or other basic estimates (e.g. regression coefficient) AND variation (e.g. standard deviation) or associated estimates of uncertainty (e.g. confidence intervals)
☐	☒	For null hypothesis testing, the test statistic (e.g. *F*, *t*, *r*) with confidence intervals, effect sizes, degrees of freedom and *P* value noted<br>*Give P values as exact values whenever suitable.*
☒	☐	For Bayesian analysis, information on the choice of priors and Markov chain Monte Carlo settings
☒	☐	For hierarchical and complex designs, identification of the appropriate level for tests and full reporting of outcomes
☒	☐	Estimates of effect sizes (e.g. Cohen's *d*, Pearson's *r*), indicating how they were calculated

Our web collection on statistics for biologists contains articles on many of the points above.

Software and code

Policy information about availability of computer code

Data collection	ELISA data were collected using a Synergy H1 plate reader (BioTek, Gen5 v.3.02 software). Flow cytometry was done using CytoFLEX S flow cytometer (Beckman Coulter). In vitro heat treatment was done in a Bio-Rad C1000 Touch thermocycler. qPCR was done in a CFX384 Touch Real-Time PCR thermocycler (BioRad). The ultrasound platform in the USgFUS setup was from Verasonics (Vantage 256, Kirkland, WA) and the small animal 1.5-MHz 128-element therapeutic array was from Imasonic (Voray sur l'Ognon, France). The imaging array was ATL L12-5 38 mm. USgFUS was controlled by a Matlab script written in the lab.
Data analysis	Flow cytometry data were analyzed with FlowJo v10. Graphpad t test calculator was used for statistical analysis.

For manuscripts utilizing custom algorithms or software that are central to the research but not yet described in published literature, software must be made available to editors and reviewers. We strongly encourage code deposition in a community repository (e.g. GitHub). See the Nature Portfolio guidelines for submitting code & software for further information.

Data

Policy information about availability of data

All manuscripts must include a data availability statement. This statement should provide the following information, where applicable:

- Accession codes, unique identifiers, or web links for publicly available datasets
- A description of any restrictions on data availability
- For clinical datasets or third party data, please ensure that the statement adheres to our policy

All data for main figures and extended figures are included in the source data file.

Human research participants

Policy information about studies involving human research participants and Sex and Gender in Research.

Reporting on sex and gender	Sex and gender are not relevant in this study. Blood were taken at random from donors through the Stanford Blood Center.
Population characteristics	Population characteristics are not relevant in this study. Blood were taken at random from donors through the Stanford Blood Center.
Recruitment	This is not relevant in this study.
Ethics oversight	Primary human T cells were isolated and cultured in vitro under a protocol approved by the Stanford University Institutional Review Board.

Note that full information on the approval of the study protocol must also be provided in the manuscript.

Field-specific reporting

Please select the one below that is the best fit for your research. If you are not sure, read the appropriate sections before making your selection.

☒ Life sciences ☐ Behavioural & social sciences ☐ Ecological, evolutionary & environmental sciences

For a reference copy of the document with all sections, see nature.com/documents/nr-reporting-summary-flat.pdf

Life sciences study design

All studies must disclose on these points even when the disclosure is negative.

Sample size	No statistical methods were used to predetermine sample size. For animal studies, three batches of animals and 4-5 animals for each batch were used with a total of 13 animals/data points. Sample sizes were determined based on relevant literature precedents.
Data exclusions	No data were excluded in this study.
Replication	All experiments were repeated with 2-5 independent biological replicates, as described in the figures and legends. For ELISA and qPCR, technical triplicates for each biological replicate were performed. All attempts at replication were successful.
Randomization	Randomization was not relevant to this study. For in vitro cell culture experiments, cells were seeded from a homogeneous suspension into 24-wells in a same volume/density. Transfection and treatment procedures were performed in the same manner to all samples. For analysis, all cells from one well were collected and mixed well for analysis. For in vivo animal treatment, same amount of cells were injected to animals while treatment and data collection were done on the same day for same batch of animals.
Blinding	Majority of the data were acquired from equipment like flow cytometry in an automated manner. No subjective bias was introduced. For in vivo animal studies, one experimentor did HIFU treatment and noted the side of tumor being treated; another experimentor collected the tumors based on animal ear tag number and a third experimentor did sample prep and flow cytometry on all the tumors. The data were when analyzed pulling information from all the experimentors.

Reporting for specific materials, systems and methods

We require information from authors about some types of materials, experimental systems and methods used in many studies. Here, indicate whether each material, system or method listed is relevant to your study. If you are not sure if a list item applies to your research, read the appropriate section before selecting a response.

Materials & experimental systems

n/a	Involved in the study
	✗ Antibodies
	✗ Eukaryotic cell lines
✗	Palaeontology and archaeology
	✗ Animals and other organisms
✗	Clinical data
✗	Dual use research of concern

Methods

n/a	Involved in the study
✗	ChIP-seq
	✗ Flow cytometry
✗	MRI-based neuroimaging

Antibodies

Antibodies used	Anti-human CXCR4 antibody was obtained from BioLegend (Clone 12G5, Cat # 306510, 1:500 dilution). Anti-human CD2 antibody was obtained from BioLegend (TS1/8, Cat # 309224, 1:50 dilution).
Validation	Validation was performed by the manufacturers. Detailed information can be found on manufacturers' product website (https://www.biolegend.com/en-us/products/apc-anti-human-cd184-cxcr4-antibody-539, https://www.biolegend.com/de-de/products/apc-anti-human-cd2-antibody-14544?GroupID=BLG10165)

Eukaryotic cell lines

Policy information about cell lines and Sex and Gender in Research

Cell line source(s)	HEK293T cells were from ATCC (Cat# CRL-3216). Jurkat cells (Cat# TIB-152) were from ATCC. Primary human T cells were isolated from donor whole blood obtained at random from the Stanford Blood Center (Stanford, CA). T cells were isolated and cultured as described in the Methods section.
Authentication	Cells used in this study were not authenticated.
Mycoplasma contamination	Cells used in this study were not tested for mycoplasma contamination.
Commonly misidentified lines (See ICLAC register)	There were no commonly misidentified lines.

Animals and other research organisms

Policy information about studies involving animals; ARRIVE guidelines recommended for reporting animal research, and Sex and Gender in Research

Laboratory animals	J:Nu female mice at 6-8 week old were purchased from Jackson Laboratory (Cat # 007850). Mice were kept at room temperature 20-24 °C with humidity 40-60% and 12 hour light/12 hour dark cycle.
Wild animals	No wild animals were used in this study.
Reporting on sex	Sex information was not relevant in this study.
Field-collected samples	No field-collected samples were used in this study.
Ethics oversight	All animal experiments were conducted under an animal use protocol approved by Stanford University, Administrative Panel on Laboratory Animal Care (APLAC) and Institutional Animal Care and Use Committee (IACUC)

Note that full information on the approval of the study protocol must also be provided in the manuscript.

Flow Cytometry

Plots

Confirm that:

- ✗ The axis labels state the marker and fluorochrome used (e.g. CD4-FITC).
- ✗ The axis scales are clearly visible. Include numbers along axes only for bottom left plot of group (a 'group' is an analysis of identical markers).
- ✗ All plots are contour plots with outliers or pseudocolor plots.
- ✗ A numerical value for number of cells or percentage (with statistics) is provided.

Methodology

Sample preparation	Samples were prepared as described in the Methods section. Briefly, cells were trypsinized (for adherent cells), resuspended in PSB+10% FBS, pipetted up and down a few times and passed through a a flow cytometry tube with a cell strainer cap (Falcon®) to get a homogeneous solution before flow cytometry. Suspension cells were pipetted and analyzed directly in their growth media. For cells expressing surface markers (i.e. CD2 and CXCR4), the staining protocol and sample preparation were detailed in the Methods section.
Instrument	CytoFLEX S flow cytometer (Beckman Coulter)
Software	FlowJo v10
Cell population abundance	At least 10,000 cells expressing proteins of interest were gated and collected, unless indicated otherwise.
Gating strategy	Samples were gated as described in the Methods section. Briefly, cells were gated for live/dead using FSC-A and SSC-A. Then single cells were gated using FSC-A and FSC-W. Then, cells were gated based on their fluorescent marker which indicates expression, as described in the Methods section.

☒ Tick this box to confirm that a figure exemplifying the gating strategy is provided in the Supplementary Information.
